# ^68^Ga-PSMA ligand PET/CT in patients with prostate cancer: How we review and report

**DOI:** 10.1186/s40644-016-0072-6

**Published:** 2016-06-08

**Authors:** Isabel Rauscher, Tobias Maurer, Wolfgang P. Fendler, Wieland H. Sommer, Markus Schwaiger, Matthias Eiber

**Affiliations:** Klinikum rechts der Isar, Department of Nuclear Medicine, Technische Universität München, Ismaninger Str. 22, 81675 Munich, Germany; Clinical Cancer Center Munich (CCM), Munich, Germany; German Cancer Consortium (DKTK), Heidelberg, Germany; Klinikum rechts der Isar, Department of Urology, Technische Universität München, Ismaninger Str. 22, 81675 Munich, Germany; Department of Nuclear Medicine, Ludwig-Maximilians University of Munich, Marchioninistrasse 15, 81377 Munich, Germany; Grosshadern Campus, Department of Clinical Radiology, Ludwig-Maximilians University of Munich, Marchioninistrasse 15, 81377 Munich, Germany

**Keywords:** Prostate cancer, Prostate specific membrane antigen, Positron emission tomography

## Abstract

Recently, positron emission tomography (PET) imaging using PSMA-ligands has gained high attention as a promising new radiotracer in patients with prostate cancer (PC). Several studies promise accurate staging of primary prostate cancer and restaging after biochemical recurrence with ^68^Ga-PSMA ligand Positron emission tomography/computed tomography (PET/CT). However, prospective trials and clinical guidelines for this new technique are still missing. Therefore, we summarized our experience with ^68^Ga-PSMA ligand PET/CT examinations in patients with primary PC and biochemical recurrence. It focuses on the technical and logistical aspects of ^68^Ga-PSMA ligand PET/CT examination as well as on the specific background for image reading discussing also potential pitfalls. Further, it includes relevant issues on free-text as well as structured reporting used in daily clinical routine.

## Background

Prostate cancer (PC) represents the most common cancer in men and accounts for the third most cause for cancer-associated death in men [1]. Early detection of primary disease and its metastases is highly relevant in terms of prognosis and therapy management. Primary staging with conventional imaging modalities such as computed tomography (CT) or magnetic resonance imaging (MRI) is limited as these techniques focus on morphologic information and LN involvement is mainly assessed by size. Up to 50 % of all patients undergoing radical prostatectomy (RP) or radiotherapy (RT) for primary treatment of PC develop biochemical recurrence [2–4]. Therefore, precise diagnosis of recurrence is crucial for patient counselling and treatment selection. However, the limited accuracy of CT or MRI in the detection of local disease in patients with biochemical recurrence is well appreciated [5, 6].

Positron emission tomography/computed tomography (PET/CT) as a hybrid imaging technique combining functional and morphological information has been proven to exhibit high diagnostic accuracy and is increasingly established as the primary staging tool in PC and in patients with suspicious recurrent disease. Several radiotracers have been proposed for molecular imaging of PC including choline as a marker of membrane cell proliferation. For recurrent PC, choline based (i.e. either ^18^F-Choline or ^11^C-Choline) PET/CT is currently widely used in clinical routine, however, there have been numerous studies reporting a low sensitivity and specificity [7, 8]. Especially in patients with prostate-specific antigen (PSA)-values below 3 ng/ml the detection rate is reported to be only 40–60 % [9–11].

Other radiotracers evaluated for PC include ^11^C-Acetate and ^18^F-FACBC. ^18^F fluciclovine, a radiolabeled leucine analog (1-amino-3-fluorocyclobutane-1-carboxylic acid in the ‘anti’ configuration [^18^F FACBC]), is used to depict amino acid transportation and has been found to be successful in the assessment of primary and metastatic PC showing also statistically significant superior detection rates in comparison to ^11^C-Choline PET [12–14]. ^11^C-Acetate is used as a PET radiotracer for imaging PC via incorporation into intracellular phosphatidylcholine membrane microdomains in cancer cells.

## Current clinical and scientific evidence for ^68^Ga-PSMA ligand PET/CT and potential indications

The prostate specific membrane antigene (PSMA) is a transmembrane protein with significantly elevated expression in PC cells compared to benign prostatic tissue. So far, several, mainly retrospective studies describe the value of ^68^Ga-PSMA ligand PET/CT in different clinical scenarios. All of them demonstrate a higher diagnostic efficacy of ^68^Ga-PSMA ligand PET/CT compared to conventional imaging including PET with other tracers (e.g. ^18^F-Choline, ^11^C-Choline) [7, 15–19]. In particular, ^68^Ga-PSMA ligand PET/CT promises accurate staging of primary PC and re-staging after biochemical recurrence. In a large study in primary intermediate to high-risk PC, ^68^Ga-PSMA-ligand imaging has been reported to clearly improve detection of lymph node metastases compared to morphological imaging thus potentially allowing for a more tailored therapeutic concept [16].

Similar encouraging results were obtained for patients with biochemical recurrence after radical prostatectomy [17]. Here, ^68^Ga-PSMA ligand PET imaging has been shown to increase detection of metastatic sites even at low PSA-values in comparison to conventional imaging or PET examination with different tracers [7]. More specifically, in a study of Afshar-Oromieh et al. ^68^Ga-PSMA ligand PET/CT detected 78 lesions characteristic for recurrent PC in 32 patients while ^18^F-fluoromethylcholine PET/CT detected only 56 lesions in 26 patients resulting in a significant higher detection rate for ^68^Ga-PSMA ligand PET/CT [7]. The advantage of ^68^Ga-PSMA ligand PET is especially evident in patients with low PSA levels (PSA below 1 ng/ml). A recent study reported a detection rate of 73 and 58 % in patients with biochemical recurrence after radical prostatectomy in a PSA-range of 0.5–1.0 ng/ml and 0.2–0.5 ng/ml, respectively [17]. This facilitates the use of salvage procedures (e.g. secondary lymphadenectomy, targeted radiation therapy) with a potentially curative intent [20]. Although ^68^Ga-PSMA ligand PET seems to have an edge over morphological imaging in patients with PC, the evaluation of PSMA-negative PC comprising around 8 % of the examined patients remains a challenge [16].

In nuclear medicine, bone imaging with ^99m^Tc-phosphonates plays an important role in the management of PC patients according to current guidelines providing a fast whole-body overview evaluating the presence of bone metastases. Preliminary results from our department indicate that the detection rate of ^68^Ga-PSMA ligand PET/CT is clearly superior to traditional bone scan [21]. It has to be mentioned that in clinical practice often faint uptake in various regions of the skeleton, especially in the ribs is found. Caution has to be taken in these cases it remains unclear whether this uptake is really related to bone metastasis or might also constitute false positive findings. Nevertheless, in cases of moderate or intense uptake (see below) usually no doubts exist concerning the presence of bone metastases. Artigas et al. recently reported about an increased ^68^Ga-PSMA ligand uptake in a patient with Paget’s disease probably related to an overexpression of PSMA in areas with an abnormal bone remodelling and increased vascularity [22]. In addition, healing fractures e.g. ribs or pelvis are known to potentially show faint increased PSMA-ligand uptake [23]. Nevertheless, currently data from multicentric prospective trials creating a high level of evidence are still missing. Therefore and due to the novelty of this technique the implementation in clinical guidelines is still missing. In addition, the reimbursement by health-care providers is broadly varying as ^68^Ga-PSMA ligand PET is still considered experimental. However, according to the opinion of the authors of this article a summary of the potential and reasonable indications for PSMA-ligand PET/CT is provided in Table 1.

**Table 1:** Potential indications for ^68^Ga-PSMA ligand PET/CT

Benefit using ^68^Ga-PSMA ligand PET/CT	Patient group
High estimated benefit/diagnostic gain	• Primary staging in high-risk disease according to D’Amico classification • Biochemical recurrence with low PSA-values (0.2 ng/ml to 10 ng/ml)^a^
Low estimated benefit/diagnostic gain	• Primary staging in low-risk (and intermediate-risk) disease according to D’Amico classification
Potential application with promising preliminary data	• Biopsy targeting after previous negative biopsy, but high suspicion of PC (esp. in combination with multiparametric MRI using PET/MRI)
Potential application with current lack of published data	• Monitoring of systemic treatment in metastatic CRPC^b^ • Monitoring of systemic treatment in metastatic castration-sensitive PC^b^ • Active surveillance (esp. in combination with multiparametric MRI using PET/MRI) • Treatment monitoring in metastatic castration-resistant PC undergoing radioligand therapy targeting PSMA (e.g. ^177^Lu-PSMA-ligand)

^a^in biochemical recurrence with PSA-values over 10 ng/ml conventional imaging (e.g. CT, MRI, bone scan) is also able to demonstrate distribution of disease. Furthermore, at PSA-values > 10 ng/ml salvage options facilitated by ^68^Ga-PSMA ligand PET/CT are unlikely

^b^Monitoring of treatment in metastasized PC patients might be enhanced due to often limited applicability of RECIST 1.1 criteria (e.g. non-target lymph node/bone metastases without extra-osseous extension) and the ineffectiveness of bone scan to reliably proving therapy response (e.g. flare phenomenon) compared to preclinical data suggesting PSMA-expression as indicator for response assessment

Furthermore, this review aims at helping physicians and physicists to execute, interpret and document hybrid ^68^Ga-PSMA ligand PET/CT examinations. It focuses on the technical and logistical issues as well as on the specific background for image reading with an emphasis on the PET-part since contrast enhanced computed tomography as the second part of a hybrid ^68^Ga-PSMA ligand PET/CT examination is an already very standardized and common imaging technique.

## Synthesis, application and imaging protocol of ^68^Ga-PSMA ligand PET/CT

A number of different PSMA-targeted PET tracers have been developed [24–27]. The most widely used PSMA-ligand for PET-imaging in Europe is a ^68^Ga-labelled PSMA inhibitor Glu-NH-CO-NH-Lys(Ahx)-HBED-CC (^68^Ga PSMA HBED-CC) followed by the theranostic agent ^68^Ga-labelled PSMA I&T [26, 27]. Details on the synthesis of ^68^Ga-labelled PSMA HBED-CC and ^68^Ga-labelled PSMA I&T have been described previously [27, 28].

The ^68^Ga-PSMA ligand complex solution is applied to patients via an intravenous bolus with an activity of 1.8–2.2 MBq per kilogram bodyweight. Variation of injected radiotracer activity is caused by the short half-life of ^68^Ga and variable elution efficiencies obtained during the lifetime of the ^68^Ge/^68^Ga radionuclide generator. Low output of the ^68^Ge/^68^Ga radionuclide generator could pose a major problem in institutions with high-throughput as it can considerable lower the number of patients that can be examined per ^68^Ga-PSMA ligand synthesis. To optimize the number of patients it is recommended to either inject patients in parallel in the case several scanners are available at one site. In addition, in our experience it seems feasible that patients with already known diffuse metastatic disease (e.g. restaging after PSMA-radioligand therapy) are injected with only 2/3 of the usual activity. In these advanced cases, the images obtained still have reasonable quality allowing for adequate judgement of therapy response.

Administration of diuretics (furosemide 20 mg) at time of tracer injection is routinely used in our department to enhance diuresis and improve image quality by reducing artifacts due to high activity of ^68^Ga-PSMA ligand in the bladder and the urinary collection system. Thus, it is recommendable to ask the patient to empty the bladder directly prior to the PET-scan. Besides no published data on the image quality in examinations with and without the application of furosemide we have seen considerable “halo artefacts” around the parts of the urinary collection systems before starting to use diuresis. This seems to be even more relevant when PET/MRI is used [29]. Additionally, all patients receive diluted oral contrast (300 mg Telebrix) and facultative a rectal filling with a negative contrast agent (100–150 mL). The latter allows better anatomical delineation of the rectum and differentiating it from adjacent structures, e.g. seminal vesicles or lymph nodes. In our department (as in most other institutions) PET acquisition is started approximately 60 min after tracer injection. However, a study of Afshar-Oromieh et al. performing ^68^Ga-PSMA ligand PET/CT 1 h and 3 h p.i concluded that although suspicious PC lesions can be evaluated with an excellent contrast as early as 1 h p.i. late images (e.g. 3 h p.i.) may be help to further clarify unclear lesions due to a better tumour to background ratio [30]. Nevertheless, it would be more complicated to fit this approach in schedule of a busy PET department, as most of the other examination (mainly ^18^F-FDG) are also started 60 min p.i.

First a diagnostic CT scan is performed from base of the skull-base to midthigh in the portal venous phase 80 s after intravenous injection of contrast agent (1.5 ml per kilogram bodyweight, e.g. Imeron 300, maximum 120 ml) followed by the PET scan. Usually the PET-acquisition starts from the midthigh to the head to exploit the reduced ^68^Ga-PSMA ligand uptake in the urinary bladder directly after voiding. In addition, this minimizes misalignment especially for the bladder which tends to be filled up during the time of the examination. Technical and logistical details for the applied diagnostic CT-examination within a hybrid PET/CT-examination are described in Boellaard et al. [31].

In our institution, all PET scans are acquired in 3D-mode with an acquisition time of 3–4 min per bed position. Emission data are corrected for randoms, dead time, scatter, and attenuation and are reconstructed iteratively by an ordered-subsets expectation maximization algorithm (four iterations, eight subsets) followed by a postreconstruction smoothing Gaussian filter (5-mm full width at one-half maximum) as applied by the different vendors. Technical details on the reconstruction and post-processing of PET-imaging data using ^68^Ga-labelled compounds are described in Baum et al. [32].

## Display of images

For image analysis all datasets are usually transferred to a dedicated postprocessing workstation (e.g. Syngo MMWP or Syngo Via, Siemens Medical Solutions, Germany; Hermes Hybrid Viewer, Hermes Medical Solutions, Sweden). The software packages for state-of-the-art PET/CT analysis allow for parallel visualisation of PET-, CT-, and PET + CT fusion images in the axial, coronal, and sagittal planes as well as maximum intensity projections in a 3D cine mode. In addition, they offer the possibility of changing the SUV-threshold to e.g. assess the uptake of PSMA-ligands in/or adjacent to organs with high background (e.g. urinary bladder, kidney). Morphological and functional imaging should be displayed at the same time and should be linked at the same table position. In addition, the diagnostic contrast-enhanced CT scan should be evaluated according to the established radiological criteria using a dedicated postprocessing workstation.

At our institution we routinely start reading the diagnostic CT scan, followed by a review of the accompanying ^68^Ga-PSMA ligand PET images with and without attenuation correction. Both uncorrected and attenuation-corrected images need to be assessed in order to identify artifacts caused by contrast agents, metal implants and/or patient motion. On all slices (of the attenuation corrected data) quantitative information with respect to size and ^68^Ga-PSMA ligand uptake can be derived. Additionally fused axial PET/CT images may be helpful for exact localization of PET positive findings.

## Pattern recognition tips/common variants and artefacts

**Fig. 1:**
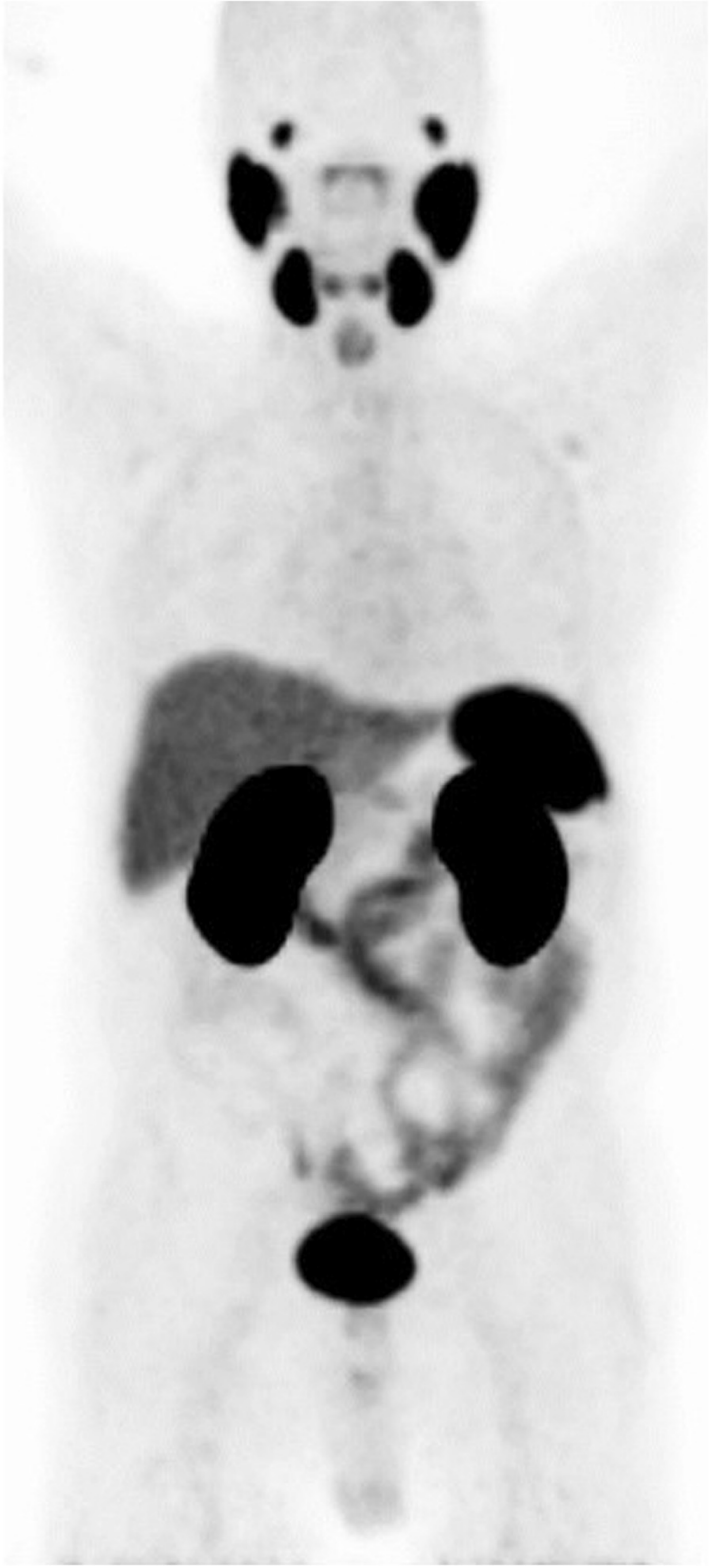
Maximum intensity protection (MIP) images of a patient with physiological distribution of ^68^Ga-PSMA ligand. Accumulation is seen in lacrimal and salivary glands, nasal mucosa, liver, spleen, bowel, kidneys and bladder
  For ^68^Ga-PSMA ligand PET interpretation, first all PET positive lesions suspicious for PC are noted. In PET any focal uptake of ^68^Ga-PSMA ligand higher than the surrounding background and not associated with physiological uptake has to be considered suspicious for malignancy and judged and described below. As ^68^Ga-PSMA ligands are excreted via the kidneys and are highly accumulated in the urinary bladder, small local recurrences might be missed. Therefore it is especially important to evaluate PET images in axial as well as coronal and sagittal planes and to change the SUV-threshold to judge the PSMA-ligand uptake in soft-tissue structures near the urinary bladder. Examples of PC patients undergoing ^68^Ga-PSMA ligand PET/CT examination for primary staging and restaging are presented in Figs. 2 and 3.

- Interpretation Criteria for ^68^Ga-PSMA ligand PET/CT
  A physiological variable PSMA-ligand uptake can be observed in the following tissues: lacrimal gland, parotid gland, submandibular gland, liver, spleen, small intestine, colon and kidney as seen in Fig. 1. Within healthy organs, kidneys and the urinary collection system including the urinary bladder as well as salivary glands are showing the highest radiotracer uptake. However, especially in localizations where PC metastases mostly occur-the retroperitoneal fatty tissue, benign lymphatic as well as bone tissue-hardly any uptake is noted [33]. Care should be taken in heavily metastasized patients as here visceral metastases can occur. Due to high background activity in the liver potential liver metastases can be obscured. In addition, in advanced disease especially liver metastases tend to loose PSMA-expression—most likely due to de-differentiation. Therefore, in advanced disease the diagnostic CT scan is the mainstay for detection of liver metastases.

**Fig. 2:**
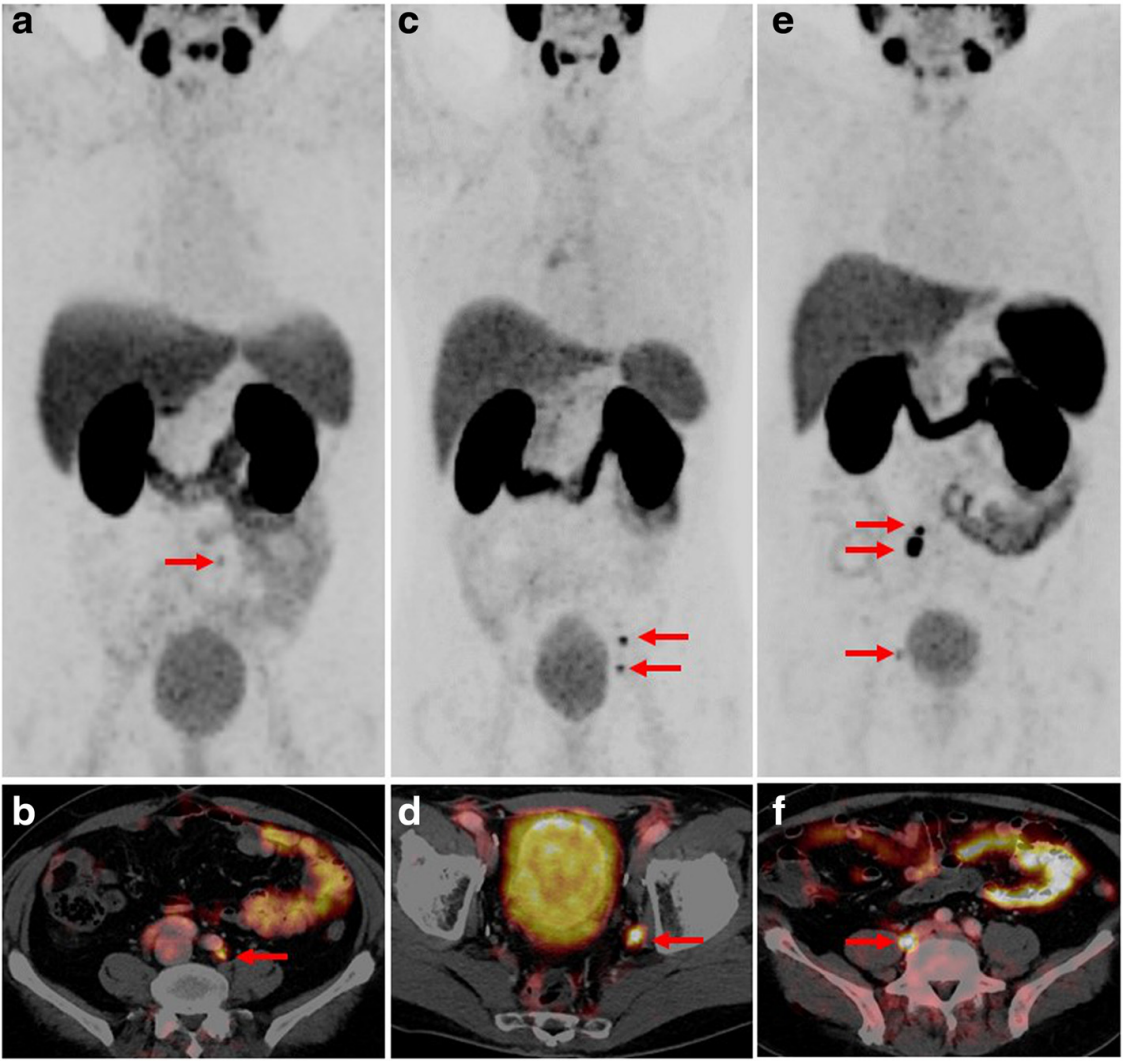
MIP **a**, **c**, **e** and fused ^68^Ga-PSMA ligand PET/CT **b**, **d**, **f** of a patient presenting with low ^68^Ga-PSMA ligand uptake in the left common iliac artery (A and B, SUV mean 3.5), a patient presenting with moderate ^68^Ga-PSMA ligand uptake in the left internal iliac artery (SUV mean 7,8) and in the left external iliac artery (C and D) and a patient presenting with intensive, focal ^68^Ga-PSMA ligand uptake behind the right common iliac vein twice (E and F, SUV mean 17,9), Note the low uptake adjacent to the urinary bladder on the right side in the MIP images (E)

**Fig. 3:**
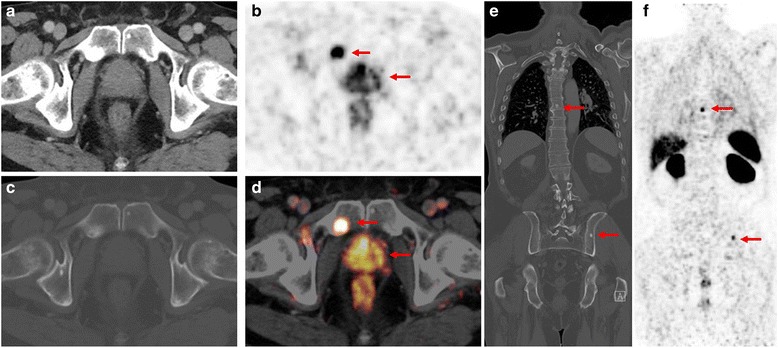
^68^Ga-PSMA ligand PET/CT in a 78-year old patient with primary PC (Gleason score 7, iPSA 34.3 ng/ml). The patient presented with intense, multifocal uptake in the prostate **b**, **d** without correlate on CT images **a** The CT examination with bone window setting **c** and **e** shows osteoblastic bone lesions in the left iliac bone as well as in the thoracic vertebrae with corresponding PSMA-ligand uptake in PET imaging **f** However, the focal PSMA-ligand uptake in the right symphysis (B, D) shows only a slight hypersclerosis (C) on corresponding CT images that might have been missed without PET. Note the physiological high uptake in the kidneys as well as moderate uptake in the liver and spleen (F)
  Finally, it has to be noted that not all PC exhibit a significant PSMA overexpression. In a study of Maurer et al. about 8 % of patients with primary PC did not show PSMA overexpression—with currently no specific biological explanation [16]. Therefore, correct and careful interpretation of the diagnostic CT scan as part of the ^68^Ga-PSMA ligand PET/CT examination is of special importance.
- Pitfalls of ^68^Ga-PSMA ligand PET
  As ^68^Ga-PSMA ligand PET/CT imaging is a relatively new imaging technique it is important to be aware that ^68^Ga-PSMA ligands are not completely specific for PC to avoid scan misinterpretation. So far, several case reports exist showing increased PSMA uptake in benign lesions such as thyroid adenoma, Paget’s disease, schwannoma, tuberculosis, adrenal adenoma or splenic sarcoidosis [22, 34–36]. Further, it is known that coeliac ganglia show a relevant ^68^Ga-PSMA uptake. In a study of Krohn et al. at least one ganglion with tracer uptake was found in 76/85 patients (89.4 %) undergoing ^68^Ga-PSMA ligand PET/CT examination which may mimic lymph node metastases in this area [37]. An example of a ^68^Ga-PSMA ligand PET/CT examination with focal uptake in the coeliac ganglion is presented in Fig. 4. Interestingly, PSMA expression has also been reported in the tumor neovasculature of some solid tumors (e.g. colon, breast, renal) and in newly formed blood vessels. Apart from PC, other malignant lesions presenting with increased PSMA expression have been reported for glioblastoma, hepatocellular carcinoma, lung cancer, renal cell carcinoma and thyroid cancer [38–42]. For example, differentiation between lung metastases and primary lung cancer which can also occur in elderly patients as secondary malignancy is a common clinical question. A study by Pyka et al. revealed that quantitative (SUV) analysis of ^68^Ga-PSMA ligand PET was not able to discriminate reliably between pulmonary metastases and primary lung cancer in PC patients as primary lung cancer lesions can also show high PSMA-expression by ^68^Ga-PSMA ligand PET. [41]. In summary, in unclear ^68^Ga-PSMA ligand PET positive lesions, morphological correlation, further clarification with other imaging techniques such as MRI, ultrasound or biopsy is mandatory.

**Fig. 4:**
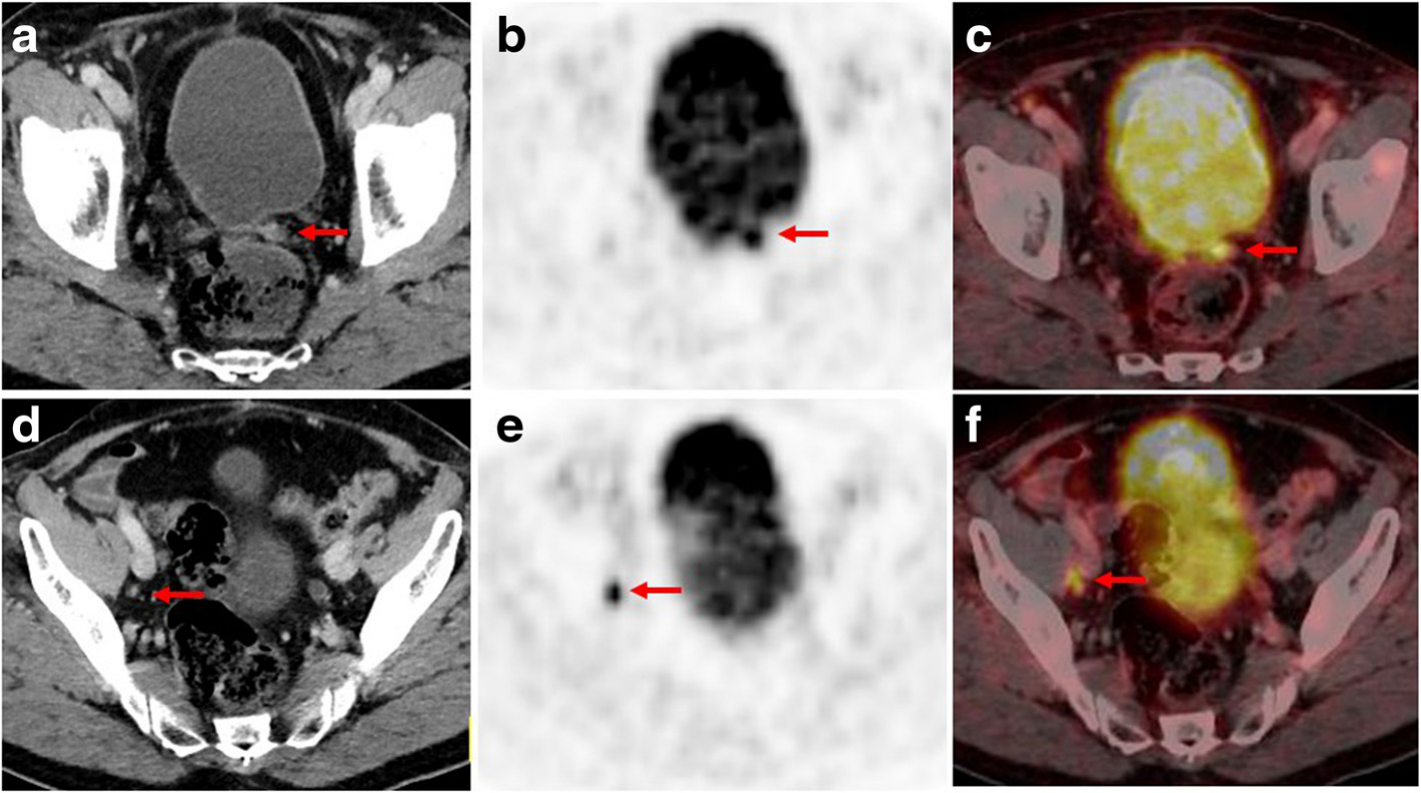
^68^Ga-PSMA ligand PET/CT examination of a 76-year old patient after radical prostatectomy (Gleason score 8, pT2b, pN1) followed by subsequent external beam radiation. 5 years later the patient presented with a rising PSA-value of 0.54 ng/ml. CT images show a 10 × 8 mm soft tissue formation in the former left prostatic bed **a** and a morphologically unsuspicious 4 mm lymph node (short axis) **d** dorsal of the right external iliac vein. Corresponding PET and fused PET/CT images show an intense, focal PSMA-ligand uptake in the former left prostatic bed **b**, **c** indicating a local recurrence as well as an intense, focal PSMA-ligand uptake in the small-sized lymph node dorsal of the right external iliac vein **e**, **f** indicating a lymph node metastasis

## Formulating reports

At first, all important clinical data and technical details of the ^68^Ga-PSMA ligand PET/CT examination should be reported in a standardized way (Table 2). Reporting of imaging findings is usually performed in a structured way on a free text basis. We recommend the following information and details to be included in the section “findings” and “conclusion”.

**Table 2:** Important clinical data and technical details of the ^68^Ga-PSMA ligand PET/CT examination

*Clinical Notes:* In patients with primary PC the following information should be included:
– date of diagnosis
– type of verification of diagnosis (biopsy results, time of biopsy, Gleason score)
– current PSA value
– previous imaging results
– relevant further diagnoses (e.g. renal insufficiency, other malignancies)
In patients with biochemical recurrence additional information is necessary:
– previous surgery and therapy (e.g. antihormonal treatment, radiation therapy, chemotherapy)
– initial PSA value, PSA nadir, facultative information on PSA-kinetics
*Technical Details:*
– exact name of the radiopharmaceutical agent and applied activity, time of scan start post injection, number of bed positions and time per bed position, body weight
– Information concerning medication administered as preparation of the PET scan (e.g. furosemide)
– CT-protocol: low-dose or/and diagnostic CT, contrast agent application (oral/rectal/ intravenous, information on concentrations and volumes, native, arterial, portalvenous), scanned portion of the body

### Findings

**Fig. 5:**
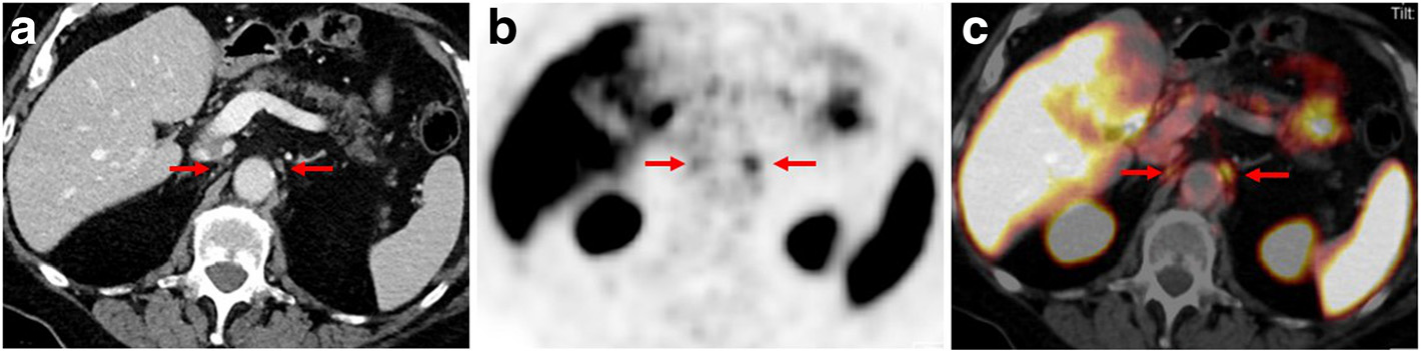
Example of a ^68^Ga-PSMA ligand PET/CT examination with incidental finding of a moderate, focal PSMA-ligand uptake in a left-sided coeliac ganglion in PET **b** and fused PET/CT images **c**. Corresponding CT images **a** show a teardrop-shaped soft tissue formation between the left adrenal gland and the truncus coeliacus measuring about 9 × 3 mm, typical for a benign coeliac ganglion. Additionally, faint PSMA-ligand uptake in projection on a smaller right-sided coeliac ganglion can be noted

Description of the localization, the extent and the intensity of pathological ^68^Ga-PSMA ligand accumulations related to normal tissue. Hereby, a standardized reporting should be pursued by reporting separately findings within the prostatic fossa, lymph nodes, bones or other visceral organs. ^68^Ga-PSMA ligand accumulation should be reported as low, moderate or intense by comparison to the background uptake in e.g. the liver parenchyma as seen in Fig. 5. For each suspicious lesion, corresponding SUVmax can be noted using a 3D isocontour VOI. However, as so far no evidence exists that specific SUV-thresholds aid in differentiation between benign and malignant lesions, at our institution quantitative SUVs are not generally reported.

Relevant findings in CT and their relation to pathological ^68^Ga-PSMA ligand accumulations should be mentioned. However, the CT part of the ^68^Ga-PSMA ligand PET/CT report must describe all pathological findings (even in the case they are PET negative).

### Conclusion

The conclusion should address the findings with respect to the clinical questions asked in the context of the ^68^Ga-PSMA ligand PET/CT examination. If possible, a definite diagnosis with regard to local tumor involvement, lymph node or bone metastases (and other if present) should be stated. Alternatively, an estimate of the probability of a diagnosis should be given. If relevant, differential diagnoses should be discussed. If appropriate, repeat examinations and/or additional examinations should be recommended to clarify or confirm findings.

However, there is growing evidence that the classical free-text reports of imaging studies have many possible flaws, like inconsistency between different readers, different nomenclature or incompleteness. Further, the narrative individual form leads to reports that do not always address key clinical questions, may contain clinically important errors and may contain ambiguous terms [43–45]. Besides, it is difficult to analyze information extracted from free-text reports.

There are large initiatives for structured reporting for different fields of imaging studies by several international societies [46]. Structured reporting assists in the clear, complete, and consistent communication of results by providing tools for physicians to adopt compliant reporting guidelines (e.g. checklists, clinical decision support). There are further efforts towards a unified reporting nomenclature (e.g. the RadLex initiative in Radiology) and towards information systems that support structured data storage and display report formats.

There have been publications for several tumor types (pancreatic cancer and rectal cancer) which showed improved satisfaction of referring physicians, higher completeness of reports when using specific report templates or checklists [47–49]. This form of reporting involves the presentation of a standard set of reporting items in a standard sequence. In urogenital radiology structured reporting is gaining more importance e.g. in the interpretation of multiparametric MRI of the prostate using the PIRADS classification [50, 51].

**Fig 6:**
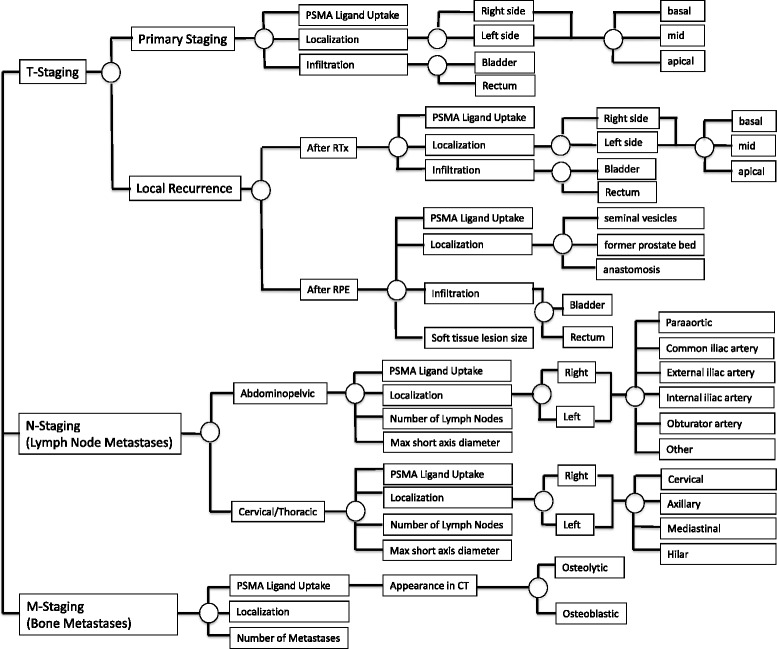
Basic template for a decision tree for structured reporting of ^68^Ga-PSMA ligand PET/CT examinations. Please note that this decision tree only contains the structured elements for local tumor involvement (primary tumor and local recurrence) as well as possible lymph node and bone metastases as the most common findings in PC. Nevertheless all other potential malignant lesions/other pathological findings have to be noted but exceed the scope of this figure

Based on these recent initiatives for structured reporting we developed a template for structured reporting of ^68^Ga-PSMA ligand PET/CT examination presented in Fig. 6. The proposed structured report divides into three categories: T-staging (primary tumor or local recurrence), N-staging (abdominopelvic or cervical/thoracic lymph nodes) and M-staging (bone). We used an online platform that allows creation of decision trees for certain examinations and concomitant report generation of semantic reports by linking text elements to the decision trees (https://www.smart-radiology.com).

## Conclusions

PET imaging for PC using PSMA-ligands has gained high attention during the last years. However, so far, no guidelines exist how to review and report ^68^Ga-PSMA ligand PET/CT examinations in patients with PC. Therefore, in this review we have summarized our approach performing ^68^Ga-PSMA ligand PET/CT scans and reviewing the images. We discussed important pitfalls in the evaluation of ^68^Ga-PSMA ligand PET/CT examinations and outlined important aspects for free-text as well as structured reports in daily clinical routine. In addition, we have outlined potential indications with an emphasis on the estimated clinical benefit.
